# dcGOR: An R Package for Analysing Ontologies and Protein Domain Annotations

**DOI:** 10.1371/journal.pcbi.1003929

**Published:** 2014-10-30

**Authors:** Hai Fang

**Affiliations:** Computational Genomics Group, Department of Computer Science, University of Bristol, Bristol, United Kingdom; University of Canterbury, New Zealand

## Abstract

I introduce an open-source R package ‘dcGOR’ to provide the bioinformatics community with the ease to analyse ontologies and protein domain annotations, particularly those in the dcGO database. The dcGO is a comprehensive resource for protein domain annotations using a panel of ontologies including Gene Ontology. Although increasing in popularity, this database needs statistical and graphical support to meet its full potential. Moreover, there are no bioinformatics tools specifically designed for domain ontology analysis. As an add-on package built in the R software environment, dcGOR offers a basic infrastructure with great flexibility and functionality. It implements new data structure to represent domains, ontologies, annotations, and all analytical outputs as well. For each ontology, it provides various mining facilities, including: (i) domain-based enrichment analysis and visualisation; (ii) construction of a domain (semantic similarity) network according to ontology annotations; and (iii) significance analysis for estimating a contact (statistical significance) network. To reduce runtime, most analyses support high-performance parallel computing. Taking as inputs a list of protein domains of interest, the package is able to easily carry out in-depth analyses in terms of functional, phenotypic and diseased relevance, and network-level understanding. More importantly, dcGOR is designed to allow users to import and analyse their own ontologies and annotations on domains (taken from SCOP, Pfam and InterPro) and RNAs (from Rfam) as well. The package is freely available at CRAN for easy installation, and also at GitHub for version control. The dedicated website with reproducible demos can be found at http://supfam.org/dcGOR.

This is a *PLOS Computational Biology* Software Article

## Introduction

Proteins are of modular design, with structural units called domains [1]. Domains often act as the operational units responsible for many aspects of protein function, and some of them are linked to phenotypic traits and diseased states. Despite their importance in biology, domains are less studied than proteins/genes in terms of ontology annotation; something much-needed and only recently addressed by the dcGO database [2]. This database provides a systematic annotation of domains using a panel of ontologies; an ontology such as Gene Ontology (GO) [3] is controlled vocabularies but organised in a hierarchy to categorise a particular sphere of knowledge. The dcGO algorithm was initially published as an improvement to the SUPERFAMILY database [4]. The quality and utility of this resource were evaluated in the Critical Assessment of Function Annotation (CAFA) competition [5], [6]. The webserver provides several mining facilities, however, web-based facilities are limited in analytical flexibility and scalability; there is a need to have a standalone tool overcoming these limitations. Currently, there are no bioinformatics tools that are specifically designed for analysing ontologies and annotations at the domain level. Most, if not all, open-source tools (such as ‘topGO’ [7], ‘GOSemSim’ [8] and ‘ontologizer’ [9]) are gene-centric and only deal with a very limited number of ontologies, usually GO. To the best of my knowledge, these tools do not provide the support for customised analysis according to users' own ontologies and annotations. To meet these needs, I have developed ‘dcGOR’, a flexible R package that provides a basic infrastructure suitable for representing ontologies and annotations. More importantly, it supports various analytical utilities tailored to this important resource. As demonstrated below, dcGOR is capable of in-depth analyses of input domains; structural bioinformatics/genomics community is increasingly confronted with this type of analysis. With this package, users are expected to understand their domains of interest: not just in the relevance to functions, phenotypes and diseases, but also at a network level. With this package, users are also able to perform customised analysis using their own ontologies and annotations.

## Design and Implementation

The dcGOR package is designed in a general way that allows for representing and analysing three bits of information: domains, ontologies and annotations. For it to be applicable in domain-centric annotations, the backend is its built-in data that is pre-compiled from the latest version of the dcGO database [2]. There are a dozen or so ontologies, such as GO, Disease Ontology (DO) [10] and Human Phenotype (HP) [11]. They are all used to annotate both SCOP domain superfamilies and families [12]. Also supported are GO annotations for domains taken from Pfam [13] and InterPro [14], and for non-coding RNAs from Rfam [15]. **Table 1** lists ontologies and annotations supported in the package.

**Table 1:** A summary of ontologies, infrastructures and functions included in dcGOR.

	Description
**Ontologies**	
***Gene Ontology***	Knowledge on functions; annotate domains from SCOP, Pfam, InterPro and RNA families from Rfam
***Disease Ontology***	Knowledge on human diseases; annotate SCOP domains only
***Human Phenotype***	Knowledge on human phenotypes; annotate SCOP domains only
***Mammalian Phenotype***	Knowledge on mouse phenotypes; annotate SCOP domains only
***Enzyme Commission***	Knowledge on enzyme activities; annotate SCOP domains only
***UniProtKB KeyWords***	Knowledge on functions and others; annotate SCOP domains only
***UniProtKB UniPathway***	Knowledge on pathways; annotate SCOP domains only
**Infrastructures**	
**InfoDataFrame**	S4 class for representing data information (e.g. domains)
***Onto***	S4 class for representing ontologies
***Anno***	S4 class for representing domain-centric annotations
***Eoutput***	S4 class for storing enrichment outputs
***Dnetwork***	S4 class for storing domain networks
***Coutput***	S4 class for storing RWR-based contact outputs
***Cnetwork***	S4 class for storing contact networks
**Functions for customised data building**	
***dcBuildInfoDataFrame***	Create an object of S4 class ‘InfoDataframe’ from an input file
***dcBuildOnto***	Create an object of S4 class ‘Onto’ from input files
***dcBuildAnno***	Create an object of S4 class ‘Anno’ from input files
**Functions for analysis and visualisation**	
***dcEnrichment***	Enrichment analysis; return an object of S4 class ‘Eoutput’
***visEnrichment***	Enrichment output visualisation
***dcDAGdomainSim***	Semantic similarity calculation; return an object of S4 class ‘Dnetwork’
***dcRWRpipeline***	Random walk with restart; return an object of S4 class ‘Coutput’
***dcDAGannotate***	Annotation propagation according to true-path rule
***dcConverter***	Conversion between different graph classes
***dcRDataLoader***	Loading RData into the current environment

The dcGOR is exclusively implemented on the R software environment. Three S4 classes are defined: ‘InfoDataFrame’ for domains, ‘Onto’ for ontologies and ‘Anno’ for annotations. The class ‘InfoDataFrame’ is used to store domain information. Since an ontology is organised as a directed acyclic graph (DAG; a directed graph without cycles), the class ‘Onto’ represents the ontology as a directed graph in which both adjacency matrix and node/term information are defined. For annotations, the class ‘Anno’ is defined to accommodate a sparse annotation matrix and additional metadata on domains and terms. All these classes have their class-specific S4 methods. This design of data representations greatly simplifies domain ontology analyses. **Table 1** outlines supported analyses: domain-based enrichment analysis, semantic similarity between pairs of annotated domains, and significance analysis for estimating a contact network.

The function *dcEnrichment* conducts enrichment analysis based on the hypergeometric/binomial distribution or Fisher's exact test [16]. It tests the statistical significance of the observed number of domains overlapped between an input group of domains and domains annotated by an ontology term. By default, all annotatable domains are used as the test background, but the user can specify this background. Taking as inputs a group of domains, *dcEnrichment* reports ontology terms that are enriched in this input domain group. To account for the ontology DAG, it also implements several algorithms that were originally applied to GO [7], [9]. The basic idea is to estimate the significance of a term after adjusting (e.g. removing) those annotations that its children terms also have. Enrichment outputs are stored as an object of S4 class ‘Eoutput’, on which methods are defined for easy view and save. Directly operating on this object, the function *visEnrichment* visualises the top significant terms in the context of the ontology DAG to aid intuitive interpretation.

Semantic similarity is a type of comparison to assess the degree of relatedness between two entities (here domains) in meaning of their annotations [17]. Semantic similarity between domains is calculated based on their annotation by ontology terms. To do so, information content (IC) of a term is defined as the negative 10-based log-transformed frequency of domains annotated to that term. This definition considers the actual usage of a term (the frequency of annotated domains it has) to measure how specific and informative the term is. The function *dcDAGdomainSim* first calculates semantic similarity between terms, which is then used to derive similarity between domains. All popular IC-based semantic similarity measures [8], [17] are supported. From pairwise term similarity, *dcDAGdomainSim* has several methods to calculate similarity between pairs of domains, including 3 best-matching (BM) based methods: average, maximum, and complete. For a term in either domain, all these BM-based methods first calculate maximum similarity to any terms in the other domain. For more detail, the reader is referred to this review [17]. The resulting domain (semantic similarity) network is stored as an object of S4 class ‘Dnetwork’, a weighted undirected graph in which domains are nodes and their semantic similarity scores as the edge weights. Notably, the higher the semantic similarity score is, the more similar the domain pair is (the edge weight). There is no hard threshold for the semantic similarity scores, but it is advisable to focus on the edges with highest weights (e.g. the top 50% of all edges).

Given a domain network (e.g. the one resulting from *dcDAGdomainSim*), the function *dcRWRpipeline* performs random walk with restart (RWR) for estimating contact strength and significance between two input groups of domains (as seeds). It is based on the earlier work [18], but has been generalised to allow for weighting domain seeds, and done so in a single step. RWR-based contact outputs are stored as an object of S4 class ‘Coutput’, including a contact (statistical significance) network that is also a weighted undirected graph (an object of S4 class ‘Cnetwork’).

In addition to the analyses above, dcGOR also has several auxiliary functions for data load, annotation propagation, graph class conversion, and fast computation. The function *dcRDataLoader* is the hub for loading all kinds of package built-in data; this simplifies data use and also makes room for the future data expansion. The function *dcDAGannotate* is supposed to propagate annotations. According to the true-path rule, a domain annotated to a term is also annotated by all its ancestor terms (propagated to the root). This ensures that only the valid part of the ontology (in terms of domain annotations) is used properly. The function *dcConverter* is able to convert an object between newly defined graph classes and the one used in packages ‘igraph’ [19] and ‘dnet’ [20]. This conversion enables network visualisation. Visualisation for pairwise semantic similarity matrix is done by package ‘supraHex’ [21]. To relieve computational burden, dcGOR utilises vectorised and parallelised operations. This high-performance parallel computing is realised via executing loops in parallel, aided by two packages ‘doMC’ and ‘foreach’.

## Results

The most common use case is to analyse a list of protein domains of interest. As a proof of principle, I use two interesting lists of domains (one from SCOP, the other from Pfam) to demonstrate the functionalities supported in the dcGOR package, particularly enrichment analysis and network analysis. Also, I show how users can benefit from this package to analyse their own domains, ontologies and annotations. All these examples are reproducible following step-by-step demos on the package website, from which results can also be found.

### Analysing SCOP domains gained in human compared to Metazoa

**Figure 1:**
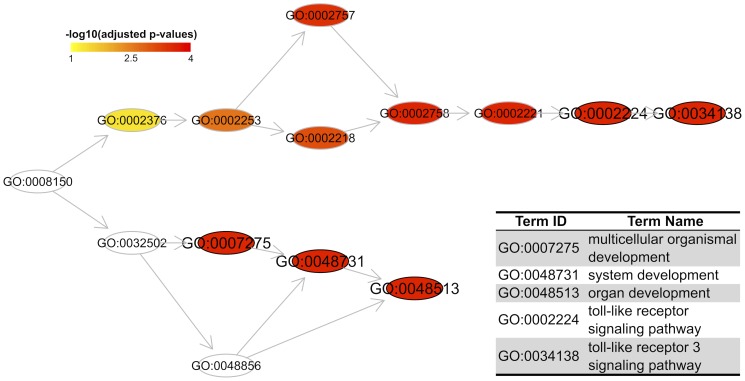
Domain-based enrichment analysis using GOBP terms. Only the most significant 5 terms/nodes (outlined in black; explained in the bottom-right panel) are visualised along with their ancestral terms. Nodes are coloured according to adjusted p-values.

First, I analyse a list of SCOP domain superfamilies that have been gained by the human genome since the ancient ancestral ‘Metazoa’ (animal). According to this report [22], a total of 1,112 SCOP domain superfamilies are present in human, among which, 58 were absent in the ancient Metazoan ancestor. Thus, these 58 domains were *de novo* gained during the evolution of the human lineage. To shed insight into these domains in the relevance to functions, phenotypes and diseases, I use *dcEnrichment* to perform enrichment analysis using all domains in Metazoa as the background. GO Biological Process (GOBP) enrichments suggest that they are of functional relevance to ‘multicellular organismal development’ and ‘toll-like receptor signalling pathway’; **Figure 1** illustrates these top enriched terms in the context of GO hierarchy. This is consistent with the fact that more complex functions evolved along the human lineage. Enrichment analysis using DO also reveals a significant link with ‘disease of cellular proliferation’.

**Figure 2:**
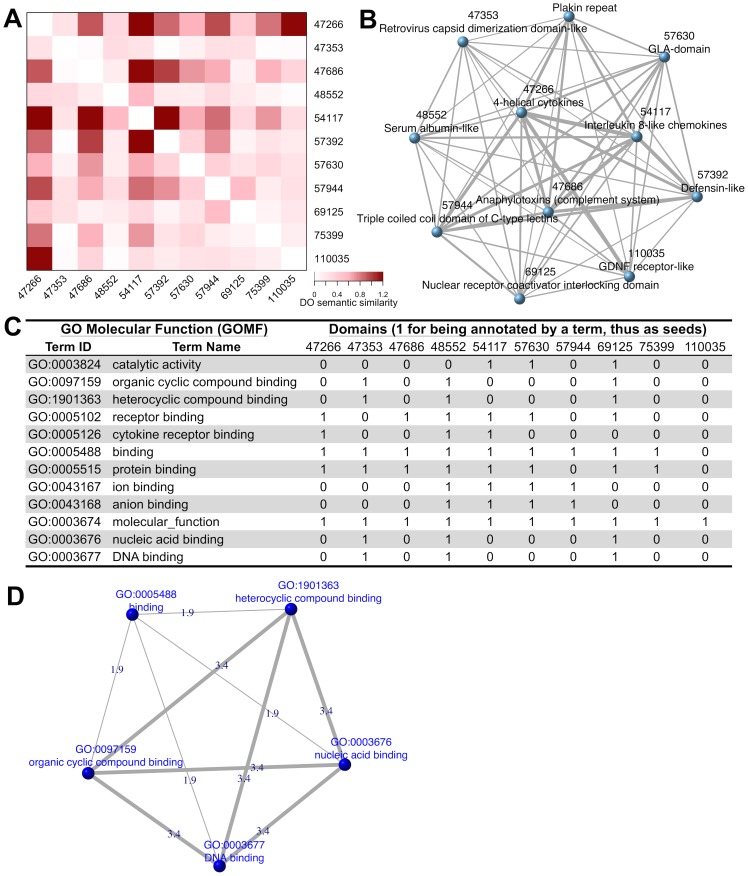
In-depth analysis for network-level understanding. (**A**) Heatmap visualisation of the semantic similarity between pairs of domains according to their annotations by Disease Ontology (DO). (**B**) Network representation of the pairwise domain semantic similarity. It is a weighted and undirected network, with edge thickness indicating semantic similarity between a pair of domains/nodes. Nodes are labeled by both numeric id and textual description. (**C**) A table listing GOMF terms and their annotated domains (used as domain seeds for random walk with restart, RWR). Notably, terms used here are only those with at least 3 annotatable domains that are also in the domain network (see Figure 2B). (**D**) Contact (statistical significance) network between GOMF terms in Figure 2C, as estimated by RWR on the domain network in Figure 2B. Only those significant contacts/edges (adjusted p-values<0.1) are shown, with thickness indicating the contact strength (z-score).

To further understand the relevance of these 58 domains to diseases, I use *dcDAGdomainSim* to construct a domain network according to domain-centric annotations by DO. This is done via calculating the semantic similarity between pairs of domains (**Figure 2A**). The resulting domain (semantic similarity) network contains 11 disease domains; they are similar to each other but to a varying degree (**Figure 2B**). Finally, based on the resultant domain network, I use *dcRWRpipeline* to estimate the contact strength and significance between sets of domains. The example domain set used here is a GO Molecular Function (GOMF) term and its annotated domains (see **Figure 2C**). The statistically significant contacts between terms are visualised in **Figure 2D**. These results suggest that (i) domains *de novo* gained during the evolution of the human lineage tend to form a disease similarity domain network, and that (ii) this network has a functional preference. Taken together, this example greatly encourages domain-centric approaches to genome evolution, function and phenotype/disease.

### Analysing promiscuous Pfam domains

**Figure 3:**
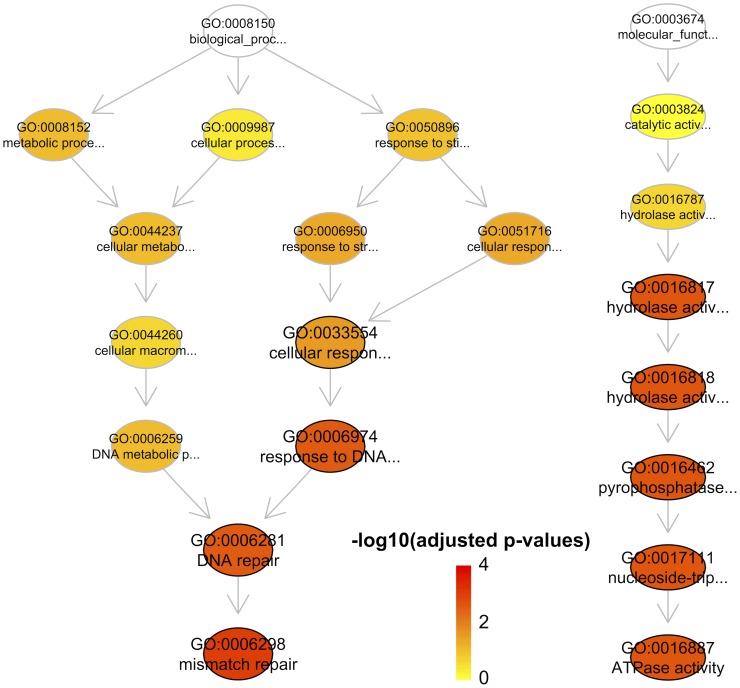
Enrichment analysis of promiscuous Pfam domains using GOBP terms (left) and GOMF terms (right). Only the most significant terms/nodes (adjusted p-values<0.05; outlined in black) are visualised along with their ancestral terms. Nodes are coloured according to adjusted p-values.

**Figure 4:**
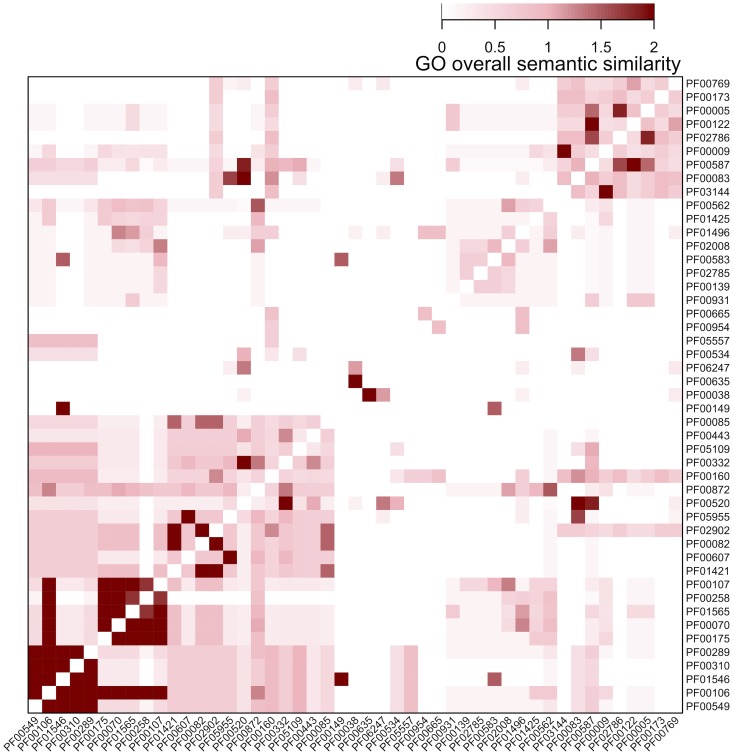
Heatmap visualisation of the GO overall semantic similarity between pairs of promiscuous Pfam domains. Domains are ordered according to hierarchical clustering by the package ‘supraHex’.

Next, I extend the analysis to a list of Pfam domains that tend to occur in diverse domain architectures; this tendency is called ‘promiscuous’. In this study [23], a total of 215 domains were identified as strongly promiscuous, in which 76 domains were taken from Pfam. Enrichment analysis of these 76 Pfam domains using GOBP terms and GOMF terms identifies two most significant terms ‘mismatch repair’ and ‘ATPase activity’ (**Figure 3**). These two functional categories are consistent with previous report, however, there is a lack of the statistical support for the relevance to ‘signal transduction’ as claimed previously [23]. Unlike DO, GO contains three sub-ontologies GOBP, GOMF and GO Cellular Component (GOCC). Therefore, the semantic similarity between pairs of these 76 domains was first calculated separately for each GO sub-ontology and then additively summed up to obtain the GO overall semantic similarity (**Figure 4**).

### Analysing users' own domains, ontologies and annotations

Unique to this package, dcGOR supports customised analysis using data files provided by users. From input files (containing relevant information on domains, ontologies and annotations), three functions (*dcBuildInfoDataFrame*, *dcBuildOnto* and *dcBuildAnno*) are able to create objects newly defined in the package (**Table 1**). Similar to the built-in data, the customised data (created objects) can be subsequently used for all analyses supported in the package. The online demo (http://supfam.org/dcGOR/demo-Customisation.html) provides detailed instructions on how to analyse (starting with input files) the InterPro2GO mapping [24].

## Availability and Future Directions

As open-source software, the dcGOR package is freely available under the GPL-2 license (see **Software S1**). For ease of installation (R package dependencies), it is distributed as part of CRAN, http://cran.r-project.org/package=dcGOR. For ease of version control, it is also distributed at GitHub, https://github.com/hfang-bristol/dcGOR. The details on documentations and demos can be found at http://supfam.org/dcGOR. As missed in most R packages, online documentations and demos are user-friendly; users can see both illustrated codes and executed outputs. This will dramatically reduce the learning curve and promote the wide adoption as users can exactly reproduce what they see.

The dcGOR is a general open-source tool for ontology and annotation analysis, providing a relatively complete framework. As demonstrated, it is able to analyse three most popular domain types (SCOP, Pfam and InterPro) and Rfam RNA families as well, and to support customised analysis. For example, users can analyse domains with different definitions, such as the partner members of the InterPro consortium [14]. The package is designed to be generic to all ontologies, not merely GO (as is the case with most existing tools) but also organism-specific ontologies. The future intention is to include those in the Open Biomedical Ontologies consortium [25]. Here I only describe a handful of analyses that are routinely required for ontology analysis, but the package is scalable for further development. Other than the data expansion aforementioned, future developments will focus on developing utilities for genome function and phenotype prediction. As the standard has been set in dcGOR, it should be much easier for ontology users/developers to extend this software to meet their needs. Also, there is no reason not to apply the similar design principles for ontology analysis at the gene level.

## Supporting Information

Software S1 Package ‘dcGOR’ (version 1.0.3) including source code, documentation and data.
(GZ) Click here for additional data file.
