# Supplementary material for: dcGOR: An R Package for Analysing Ontologies and Protein Domain Annotations
Source: PLoS Comput Biol. 2014 Oct 30;10(10):e1003929. doi: 10.1371/journal.pcbi.1003929 (PMC4214615; doi:10.1371/journal.pcbi.1003929)
Supplement: Software S1 — Package ‘dcGOR’ (version 1.0.3) including source code, documentation and data. (GZ) [file pcbi.1003929.s001.gz › dcGOR/inst/staticdocs/templates/navbar.html]

{{#package}}{{package}} {{version}}{{/package}}

- Home

- Installation

- Documentations

- Demos

- Citation
