# Supplementary figures and images for: dcGOR: An R Package for Analysing Ontologies and Protein Domain Annotations
Source: PLoS Comput Biol. 2014 Oct 30;10(10):e1003929. doi: 10.1371/journal.pcbi.1003929 (PMC4214615; doi:10.1371/journal.pcbi.1003929)

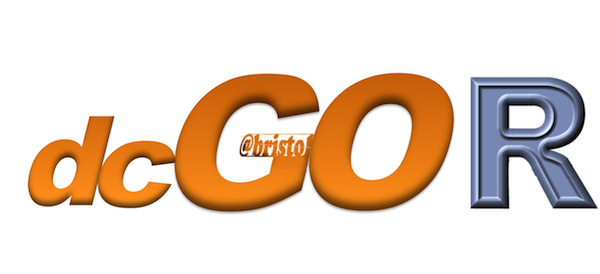

Supplement: Software S1 — Package ‘dcGOR’ (version 1.0.3) including source code, documentation and data. (GZ) [file pcbi.1003929.s001.gz › dcGOR/inst/staticdocs/images/dcGOR_logo.png]
